# Expression of nuclear factor kappa B in ovine maternal inguinal lymph nodes during early pregnancy

**DOI:** 10.1186/s12917-022-03373-7

**Published:** 2022-07-11

**Authors:** Leying Zhang, Taipeng Zhang, Zhen Yang, Chunjiang Cai, Shaopeng Hao, Ling Yang

**Affiliations:** Department of Animal Science, School of Life Sciences and Food Engineering, Hebei University of Engineering, No. 19 Taiji Road, Handan, 056038 China

**Keywords:** Lymph node, Nuclear factor kappa B, Pregnancy, Sheep

## Abstract

**Background:**

Pregnancy-induced immunological changes contribute to the maternal immune tolerance. Nuclear factor kappa B (NF-κB) pathway participates in regulating both innate and adaptive immunities, and lymph nodes play key roles in adaptive immune reaction. However, it is unclear whether early pregnancy changes the expression of NF-κB family in maternal lymph node in sheep.

**Methods:**

In this study, the samples of inguinal lymph nodes were collected from ewes on day 16 of the estrous cycle, and on days 13, 16 and 25 of pregnancy, and expression of NF-κB family, including NF-κB p105 (*NFKB1*), NF-κB p100 (*NFKB2*), p65 (*RELA*), RelB (*RELB*) and c-Rel (*REL*), were analyzed through real-time quantitative PCR, Western blot and immunohistochemical analysis.

**Results:**

The expression levels of NF-κB p105 and c-Rel downregulated, but NF-κB p100 upregulated on day 25 of pregnancy. The expression levels of p65, RelB and c-Rel peaked at day 13 of pregnancy, and expression level of RelB was higher during early pregnancy comparing to day 16 of the estrous cycle. In addition, p65 protein was located in the subcapsular sinus and lymph sinuses.

**Conclusion:**

This paper reported for the first time that early pregnancy has effects on the expression of NF-κB family, which may contribute to the maternal immunoregulation through blood circulation and lymph circulation during early pregnancy in sheep.

**Supplementary Information:**

The online version contains supplementary material available at 10.1186/s12917-022-03373-7.

## Background

There is an approximation of genetically discordant maternal and fetal tissues during pregnancy, and pregnancy-induced immunological changes may understand this immune tolerance [1]. The fetus evades maternal immune detection and elimination under the effects of high concentration of circulating progesterone (P4) and conceptus signals on the maternal immune function [2]. The trophectoderm cells of ruminant conceptuses secrete interferon tau (IFNT, the pregnancy recognition signal) that prevents luteolysis to allow corpus luteum continuous release P4 [3]. IFNT and P4 regulate gene expression of uterus during the peri-implantation period of pregnancy, which are essential for fetal growth, development, and survival [4]. IFNT also has extra-uterine functions, and induces expression of interferon stimulated genes (ISGs) in corpus luteum, the liver [5], intra-hypothalamus and anterior pituitary [6], bone marrow [7], the thymus [8], and the spleen [9, 10] in an endocrine manner. In addition, there is an upregulation of ISGs in inguinal lymph nodes during early pregnancy in ewes [11, 12]. Therefore, the effects of early pregnancy include at least the actions of IFNT, and P4. It is presumptive that early pregnancy may change the functions of maternal inguinal lymph nodes, which is associated with the maternal tolerance to fetal antigen [11, 12].

Lymph nodes disperse throughout the body, and function as key sites for the initiation of adaptive immune responses and immune surveillance [13]. The weights of the lymph nodes increase during pregnancy in rats and mice [14, 15]. Analysis of paraaortic lymph node cells indicates that the specific alloreactivity is downregulated in the pre-implantation and implantation stages of pregnancy, but the specific and non-specific alloreactivities are upregulated at mid-pregnancy [16]. Early pregnancy also changes expression of P4 receptor, P4-induced blocking factor [17], prostaglandin synthases [8], **T-helper** cytokines in maternal inguinal lymph nodes of ewes [18]. In addition, expression of melatonin receptor 1, CD4, gonadotropin releasing hormone (GnRH) and GnRH receptor is changed in maternal inguinal lymph nodes during early pregnancy in sheep [19, 20]. It is suggested that early pregnancy may have effects on immunoregulatory signaling pathway, for example nuclear factor kappa B (NF-κB) pathway [21].

NF-κB pathway plays a central role in regulating both innate and adaptive immunities, and NF-κB family includes NF-κB p105 (*NFKB1*), NF-κB p100 (*NFKB2*), p65 (*RELA*), RelB (*RELB*) and c-Rel (*REL*) [22]. Many extracellular and intracellular signals, including tumor necrosis factor (TNF), Toll-like receptors (TLRs) and various cells stressors, trigger NF-κB activation [23]. There is an inhibitory interaction between estrogen receptors and p65, which is involved in estrogen action in pregnancy [24]. NF-κB pathway is implicated in implantation and spiral artery remodeling through regulation of cytokine expression, but excessive increased NF-κB activation results in uteroplacental dysfunction and development of preeclampsia in rats [25]. Pregnancy has negative effects on the expression of NF-κB, and serum P4 also inhibits NF-κB expression in rat laryngeal mucosa [26]. NF-κB p65 level of maternal blood is higher in the women with chorioamnionitis comparing with normal women, which can be used to predict subclinical chorioamnionitis [27]. There is an upregulation of NF-κB during implantation, and then NF-κB expression is decreased for pregnancy maintenance [28]. It is hypothesized that early pregnancy may change expression of NF-κB family. Therefore, the objective of this study was to explore the expression of NF-κB p105 (*NFKB1*), NF-κB p100 (*NFKB2*), p65 (*RELA*), RelB (*RELB*) and c-Rel (*REL*) in maternal inguinal lymph nodes during early pregnancy in sheep.

## Results

### Relative expression values of *NFKB1*, *NFKB2*, *RELA*, *RELB* and *REL* mRNA in maternal inguinal lymph nodes

**Fig. 1:**
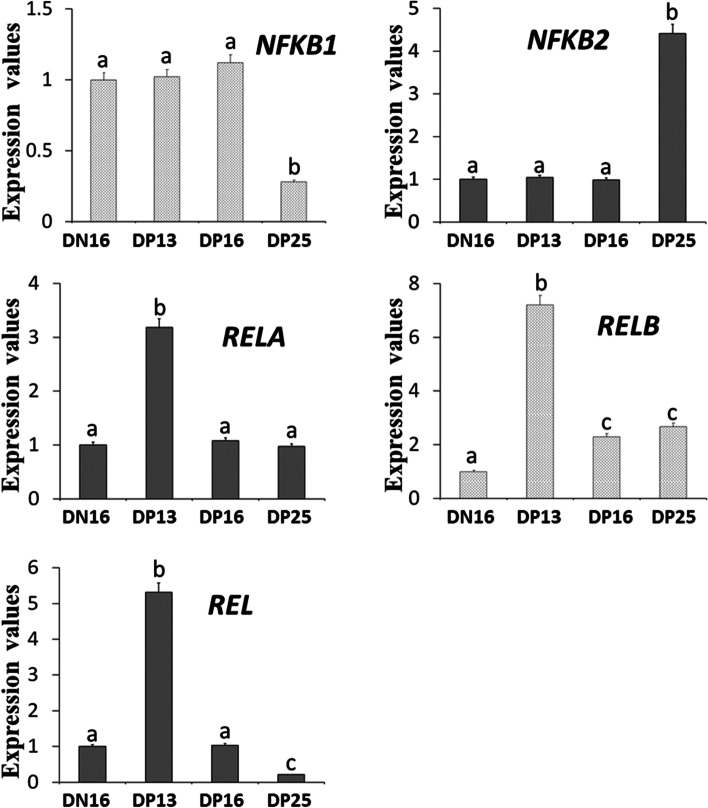
Relative expression values of *NFKB1*, *NFKB2*, *RELA*, *RELB* and *REL* mRNA in the inguinal lymph nodes from non-pregnant ewes and pregnant ewes (*n* = 6 for each group). The mean mRNA expression level for each target gene was normalized to the expression of glyceraldehyde-3-phosphate dehydrogenase (*GAPDH*), and the mean ΔCt value for the tissues collected from the cyclic ewe was a calibrator to compare the expression values from different stages of gestation. Note: DN16 = day 16 of the estrous cycle; DP13 = day 13 of pregnancy; DP16 = day 16 of pregnancy; DP25 = day 25 of pregnancy. Significant differences (*P <* 0.05) are indicated by different letters

The RT-qPCR assay showed that the relative expression level of *NFKB1* mRNA was lower on day 25 of pregnancy (DP25) compared to day 16 of the estrous cycle (DN16), and days 13 and 16 of pregnancy (DP13 and DP16) in the inguinal lymph nodes (*P* < 0.05), but the level of *NFKB2* mRNA was higher on DP25 compared to DN16, DP13 and DP16. The levels of *RELA*, *RELB* and *REL* mRNA were the highest on DP13 among the four groups, but there was no significant difference in the *RELA* expression level among DN16, DP16 and DP25 (*P* > 0.05). Furthermore, the relative expression level of *RELB* mRNA was higher on DP16 and DP25 than DN16 (*P* < 0.05), and expression level of *REL* mRNA was lower on DP25 than DN16 and DP16 (*P* < 0.05; Fig. 1).

### Expression of NF-κB p105, NF-κB p100, p65, RelB and c-Rel proteins in maternal inguinal lymph nodes

**Fig. 2:**
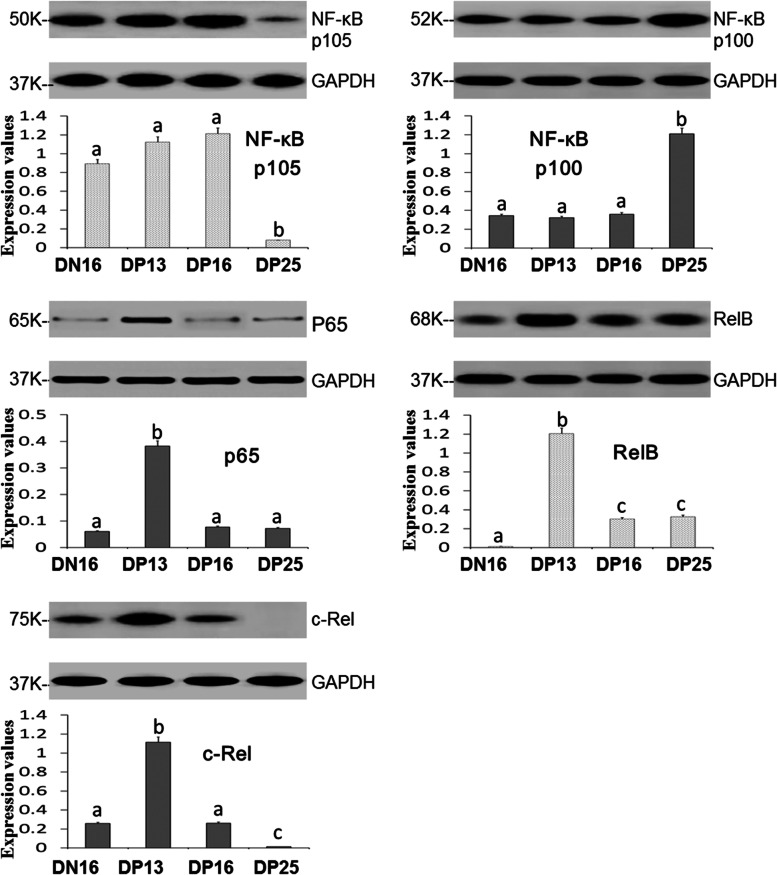
Expression of NF-κB p105, NF-κB p100, p65, RelB and c-Rel proteins in the inguinal lymph nodes from non-pregnant ewes and pregnant ewes (*n* = 6 for each group). The mean band intensity was normalized to the band intensity of GAPDH protein, and the relative protein expression level for each target protein from the cyclic ewe was a calibrator to compare the expression values from different stages of gestation. Note: DN16 = day 16 of the estrous cycle; DP13 = day 13 of pregnancy; DP16 = day 16 of pregnancy; DP25 = day 25 of pregnancy. Significant differences (*P <* 0.05) are indicated by different letters within the same color column

Western blot analysis showed (Fig. 2) that there was a downregulation of NF-κB p105 and c-Rel proteins on DP25 comparing to DN16, DP13 and DP16 (*P* < 0.05), but the expression level of c-Rel protein was higher on DP13 comparing to DN16 and DP16 (*P* < 0.05). The expression of NF-κB p100 protein was significantly upregulated on DP25 comparing to other three groups (*P* < 0.05). The expression of p65 and RelB proteins peaked at DP13 (*P* < 0.05), and RelB protein level was higher on DP16 and DP25 than DN16 (*P* < 0.05). In addition, there was no significant difference in the expression level of p65 protein among DN16, DP16 and DP25 (*P* > 0.05).

### Immunohistochemistry for p65 protein in maternal inguinal lymph nodes

**Fig. 3:**
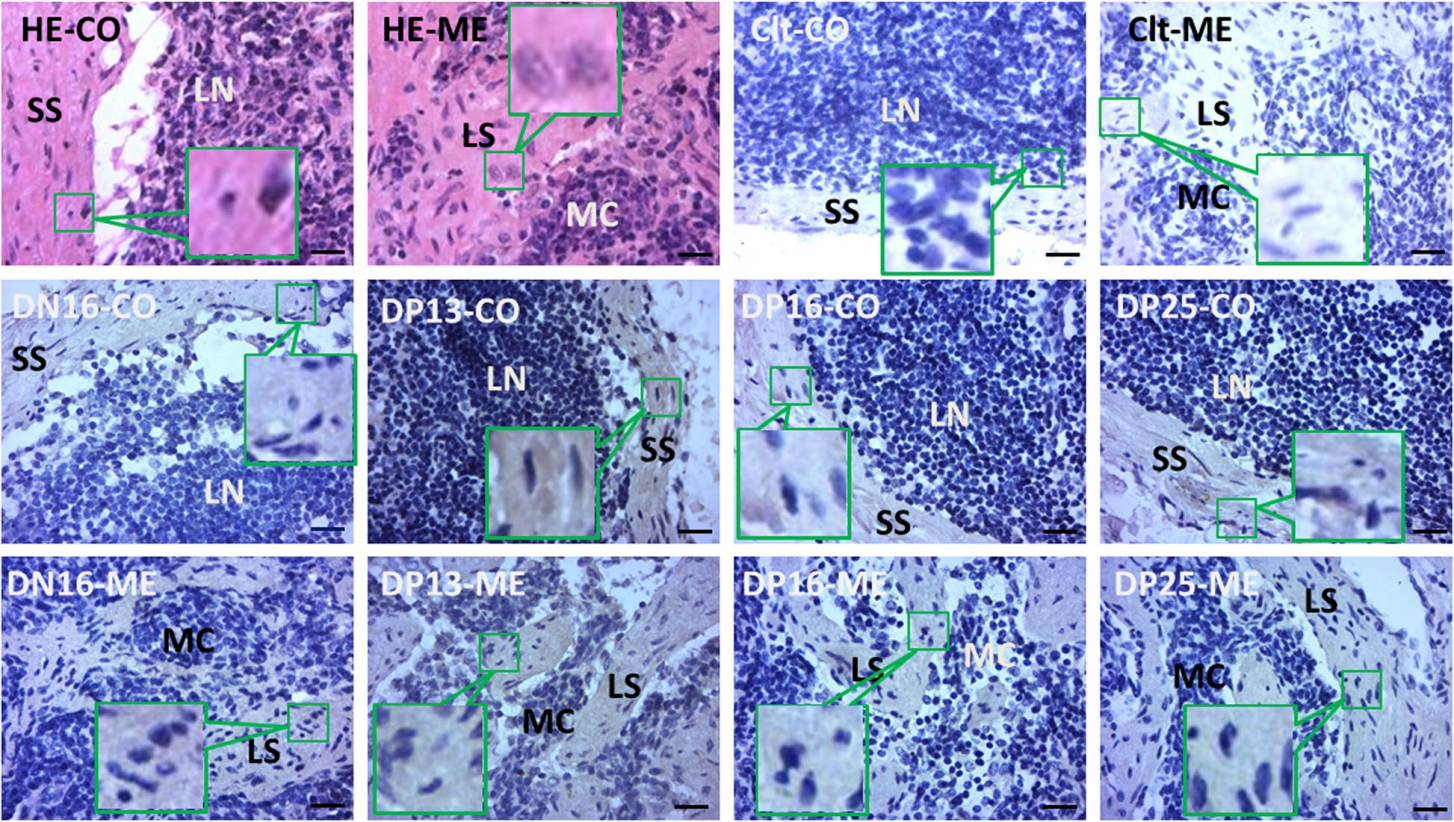
Representative immunohistochemical localization of p65 protein in the inguinal lymph nodes from non-pregnant ewes and pregnant ewes (*n* = 6 for each group). Lymph node is divided into an outer cortex (CO) and an inner medulla (ME). Lymph enters the convex through the subcapsular sinus (SS) and trabeculae (TR) around the lymphoid nodules (LN), and flows into the medulla through the lymph sinus (LS) around the medullary cord (MC). Note: HE = stained by haematoxylin and eosin; Clt = Negative control; DN16 = day 16 of the estrous cycle; DP13 = day 13 of pregnancy; DP16 = day 16 of pregnancy; DP25 = day 25 of pregnancy. Bar = 20 μm

The p65 protein was located in the subcapsular sinus and lymph sinuses, but there was almost no immunostaining in lymphoid nodule and medullary cords (Fig. 3). The staining intensities for p65 were 0, 1+, 2+, 1+, and 1+ for the negative control, the inguinal lymph nodes from DN16, and inguinal lymph nodes from DP13, DP16, and DP25, respectively (Fig. 3). The staining intensity was as follows: 0 = negative; 1+ = weak; 2+ = strong.

## Discussion

Our results revealed that mRNA and protein of NF-κB p105 downregulated in the inguinal lymph nodes at DP25 comparing to the other three groups, which may attenuate inflammatory responses. NF-κB p105 is a subunit of NF-κB complexes, and plays a role in regulating NF-κB activity and inflammatory diseases [29]. The level of NF-κB p105 in cultured monocytes exposed to the preeclamptic plasma is higher than that exposed to the plasma from normotensive pregnancy, which leads to upregulation of proinflammatory cytokine interleukin 1α (IL-1α), IL-6, and TNF-α [30]. NF-κB p105 level is downregulated in myometrium of pregnant women compared with nonpregnant controls [31].

Our data indicated that *NFKB2* mRNA and NF-κB p100 protein were upregulated in the inguinal lymph nodes at DP25 comparing to the other three groups, and the upregulation of NF-κB p100 in maternal inguinal lymph nodes may be associated with the pregnancy advances in ewes. Activation of NF-κB p100 subunit is mediated by non-canonical NF-κB signaling to regulate specific immunological processes through inhibition of the degradation of IkappaB-alpha in the canonical NF-κB pathway [32]. There is an increase in maternal plasma corticotropin-releasing hormone (CRH) abundance as pregnancy advances, which is mediated by RelB/NF-κB p100 in human placenta [33]. Signal transducer and activator of transcription 3 participates in constitutively activation of RelB/NF-κB p100 to regulate pro-labor genes in the human placenta [34]. The activation non-canonical NF-κB signaling (RelB/NF-κB p100) induces expression of CRH and prostaglandin-endoperoxide synthase-2 in the human placenta, which leads to initiating parturition in humans [35].

In this study, the levels of *RELA* mRNA and p65 protein increased at DP13, and then declined at DP16 and DP25. In addition, the p65 protein was located in the subcapsular sinus and lymph sinuses. The above finding may support the idea that peak of p65 expression level at DP13 in maternal inguinal lymph nodes is related to early pregnancy through lymphatic circulation, and the downregulation at DP16 and DP25 is favorable for pregnancy success. As a member of NF-κB family, p65 subunit is a critical regulator of the cellular localization and functions of NF-κB, which controls transcriptional competence of NF-κB [36]. There is an upregulation of endometrial p65 in the women with recurrent implantation failure, which indicates that high level of endometrial p65 is unfavorable for pregnancy success in humans [37]. The p65 level in isolated T cells is suppressed during pregnancy, which leads to inhibition of interferon γ and IL-2, suggesting that the loss of cytokine production is favorable for pregnancy success [38]. NF-κB p65 level in the isolated T cells from peripheral blood mononuclear cells is downregulated in pregnant females, which is necessary for the suppression of Th1 cytokine production and pregnancy maintenance [39]. Lymph and migrating cells enter the cortex through subcapsular sinus, and flows into the medulla through lymph sinus [40].

Our results demonstrated that expression levels of *RELB* mRNA and RelB protein peaked in maternal inguinal lymph nodes at DP13, and then declined at DP16 and DP25. It was proposed that the upregulation of RelB may be associated with embryo implantation, and have no adverse effects on normal pregnancy owing to low responsiveness to the canonical pathway. Glucocorticoid receptor signaling is implicated in constitutive activation of the noncanonical NF-κB pathway in term human placenta, which is related with upregulation placental CRH and RelB [41]. Women exposed to phthalate increase risk for delivering preterm, and the mechanism is that phthalate induces upregulation of *CRH* and cyclooxygenase 2 mRNA and protein mediated by RelB/p52 in primary culture of cytotrophoblast [42]. Decidual endothelial cells have a strong constitutive RelB activation (the non-canonical NF-κB pathway), but low responsiveness of the canonical pathway to lipopolysaccharide, which can avoid pregnancy failure [43]. RelB is involved in lymphocyte development, which is related to the production of immunoglobulins and specific antibodies in humans [44].

In this study, *REL* mRNA and c-Rel protein were peaked at DP13, and then declined in maternal inguinal lymph nodes during early pregnancy. It was suggested that the high level of c-Rel at DP13 may be related with implantation, and downregulation of c-Rel at DP16 and DP25 may be helpful for pregnancy maintenance. NF-κB subunit c-Rel plays an important role in initiation of inflammatory responses, but also functions as a transcriptional repressor of inflammatory genes [45]. NF-κB subunit c-Rel expresses in villi of normal placenta, and plays a key role in enhancing the invasion of choriocarcinoma cells through phosphatidylinositol-3-OH kinase-Akt signaling [46]. Low levels of NF-κB subunits c-Rel, p50, and p105 in myometrium are favorable for pregnancy maintenance, but increasing expression of p65, c-Rel, and p50 subunits is associated with the onset of parturition in humans [29]. Interactions between NF-κB subunits p65, RelB, c-Rel, p50 and p52 are involved in regulation of pro-inflammatory mediators in the human myometrium during pregnancy and parturition [47].

## Conclusion

Early pregnancy changed expression of NF-κB p105, NF-κB p100, p65, RelB and c-Rel in maternal inguinal lymph nodes, and p65 protein was located in the subcapsular sinus and lymph sinuses. Therefore, it may be via blood circulation and lymph circulation that early pregnancy has effects on the subcapsular sinus and lymph sinuses to regulate expression of NF-κB family, which may be related to the immunoregulation of maternal inguinal lymph nodes in sheep.

## Materials and methods

### Animals and experimental design

The experiments were performed on healthy and cyclic Small-tail Han ewes (16 to 20 months of age), and the ewes were housed under the same environmental conditions with free access to water and mineral licks. Estrus was detected using caudaepididyectomized rams, and 24 ewes were randomly divided into four groups (ewes from DN16, and ewes from DP13, DP16 and DP25, *n* = 6 for each group). After detection of sexual receptivity (day 0), the ewes of groups DP13, DP16 and DP25, but not of DN16 were mated by 2 intact rams twice at 12-h interval. Inguinal lymph nodes reside within the femoral triangle, and lymphatic drainage of the uterus and ovaries is via inguinal lymph nodes. Inguinal lymph nodes were sampled from the ewes on the morning of each indicated days (DN16, DP13, DP16 and DP25) after slaughter. Pregnancy was confirmed through observing presence of an apparently normal conceptus in uterus. The tissues (0.3 cm^3^) of inguinal lymph nodes were rinsed in paraformaldehyde in phosphate-buffered saline (PBS), and then fixed in fresh 4% paraformaldehyde in PBS. The remaining portions of lymph samples were snap-frozen in liquid nitrogen for subsequent RNA and protein extraction.

### RNA extraction and RT-qPCR assay

Approximately 20 mg of inguinal lymph node tissue were homogenized into powder in liquid nitrogen using a sterile mortar and pestle, and total RNA was obtained using TRIzol reagent (Invitrogen, Carlsbad, CA, USA) according to the manufacturer’s instructions. The RNA purity was evaluated by agarose gel (1%) electrophoresis, and optical density at 260/280 nm was in the range of 1.8 and 2.1. RNA integrity was assessed by examining the 28S and 18S rRNA bands of representative samples. Genomic DNA was eliminated, and total RNA (1 μg) was reverse transcribed into cDNA using a FastQuant RT kit (With gDNase, KR106, Tiangen Biotech Co., Ltd., Beijing, China) according to the manufacturer’s recommendations. The specific primers (Table 1) corresponding to the target genes and reference gene (*GAPDH*) were designed and synthesized by Shanghai Sangon Biotech Co., Ltd., (Shanghai, China), and assessed by BLAST (https://blast.ncbi.nlm.nih.gov/Blast.cgi) at NCBI. PCR amplification efficiency of each pair of primers was assessed before quantification, and was found to be in an acceptable range (between 0.9 and 1.1). The primer product was sequenced to check for specificity, and the melting curve was analyzed to guarantee the specificity of the amplification after PCR reactions. Amplification conditions consisted of 95 °C for 10 sec, 60–62 °C (60 °C for *NFKB1* and *NFKB2*, 61 °C for *REL*, and 62 °C for *RELA* and *RELB*) for 20 sec, and 72 °C for 25 sec, and the number of PCR cycle was 40. The data were analyzed using the 2^-ΔΔCt^ method as described previously [48]. The mean mRNA expression level for each target gene in each sample was normalized to the expression of *GAPDH*, and was expressed relative expression to the calibrator sample. The mean ΔCt value for the tissues collected from the cyclic ewes as a calibrator to compare the changes in gene expression levels among the tissues isolated from different stages of gestation.

**Table 1:** Primers used for RT-qPCR

Gene	Primer	Sequence	Size (bp)	Accession numbers
*NFKB1*	Forward	CAAGCACAAGAAGGCAGCACAAC	113	XM_027970852.2
	Reverse	CAGCCATCAGCAGCAGCAGAC		
*NFKB2*	Forward	GCCTGCTGAATGCCCTGTCTG	146	XM_042238744.1
	Reverse	CTCTGTTTCCTGTTCCACCGACTG		
*RELA*	Forward	TGGCGAGAGGAGCACAGACAC	92	XM_027959295.2
	Reverse	TGACCAGGGAGATGCGGACTG		
*RELB*	Forward	CGCTGACCTCTCCTCGCTCTC	93	XM_015100238.3
	Reverse	AAGCCGAAGCCATTCTCCTTGATG		
*REL*	Forward	TCCTCCTCTGCGTCCATCTCAAG	104	XM_004005929.4
	Reverse	GTGGGGTGGGCGATTGATGAC		
*GAPDH*	Forward	GGGTCATCATCTCTGCACCT	176	NM_001190390.1
	Reverse	GGTCATAAGTCCCTCCACGA		

### Western blot analysis

The total proteins were extracted using RIPA lysis buffer (BL504A, Biosharp, Hefei, China), and a BCA protein assay kit (Tiangen Biotech) was used to determine concentrations of total proteins with bovine serum albumin as the standard. The protein samples (10 μg/lane) were resolved on 12% SDS-PAGE gels, and then were transferred to immun-blot polyvinylidene difluoride membranes (Millipore, Bedford, MA, USA). Membranes were incubated with a mouse anti-NF-κB p105 monoclonal antibody (Santa Cruz Biotechnology, Inc., sc-8414, 1:1000), a mouse anti-NF-κB p100 monoclonal antibody (Santa Cruz Biotechnology, sc-7386, 1:1000), a mouse anti-p65 monoclonal antibody (Santa Cruz Biotechnology, sc-8008, 1:1000), a mouse anti-RelB monoclonal antibody (Santa Cruz Biotechnology, sc-166,416, 1:1000), and a mouse anti-c-Rel monoclonal antibody (Santa Cruz Biotechnology, sc-6955, 1:1000) at 4 °C overnight, respectively. The specificities of primary antibodies were confirmed through peptide blocking experiments, and the antibodies were specific for the ovine proteins. Primary antibodies were identified using a horseradish peroxidase (HRP)-conjugated secondary antibody (goat anti-mouse, Biosharp, BL001A, 1:5000). An anti-GAPDH antibody (Santa Cruz Biotechnology, sc-20,357, 1:1000) was used as an internal control protein, and a pro-light HRP chemiluminescence kit (Tiangen Biotech) was used to detect HRP-labeled secondary antibody. The band intensities were digitally quantified using the Quantity One V452 (Bio-Rad Laboratories, Hercules, CA, USA). The mean band intensity for each target protein in each sample was normalized to the band intensity of GAPDH protein, and is expressed relative expression to the calibrator sample. The relative protein expression level for each target protein from the cyclic ewes as a calibrator was to compare the changes in protein expression levels from different stages of gestation.

### Immunohistochemical analysis

Immunohistochemistry was carried out according to a previous study [11]. After fixation, the lymphatic tissue was dehydrated with increasing concentrations of ethanol, clarified in xylene, and embedded in paraffin. Sections were mounted on glass slides after sectioning. Some slides were stained by haematoxylin and eosin. Other slides were incubated in 0.01 M citrate buffer for antigen retrieval after deparaffinization and rehydration. Endogenous peroxidase activity was prevented by incubation with 3% hydrogen peroxide, and nonspecific binding sites was blocked with 5% normal goat serum diluted in PBS. Subsequently, the slides were incubated in a humidified chamber with the primary antibody specific to p65 (Santa Cruz Biotechnology, Inc., sc-8008, 1:200). Thereafter, the sections were incubated with the anti-mouse IgG-HRP (Biosharp, BL001A) antibody diluted at 1:2000. Protein localization was demonstrated with a DAB kit (Tiangen Biotech) according to the manufacturer’s instructions, and the sections were counterstained with hematoxylin. Negative controls underwent all steps with antiserum-specific isotype instead of the primary antibody at the same protein concentration. The slides were examined using a microscope (Nikon Eclipse E800, Tokyo, Japan) with digital camera DP12 under 400× magnification. Images obtained from the microscope were analyzed independently through the images by 4 observers, and the immunostaining for p65 protein was classified by assigning an immunoreactive intensity of a scale of 0 to 3 in a blinded fashion, as described previously [49].

### Statistical analysis

The RT-qPCR and Western blot results were analyzed with least-squares ANOVA using the general linear model procedures of the Statistical Analysis System Package version 9.1 for Windows (SAS Institute, Cary, NC, USA). The data were averaged to calculate the means (± standard error of the mean), which were used for comparisons among groups using one-way analysis of variance followed by a Bonferroni post hoc test after testing for normality. Each group consisted of six replicates. All differences with *p* values < 0.05 were considered statistically significant.

## Supplementary Information

**Additional file 1.**

## Data Availability

All data generated or analyzed during this study are included in this published article.

## Abbreviations

CRH: Corticotropin-releasing hormone
DN16: Day 16 of the estrous cycle
DP13: Day 13 of pregnancy
DP16: Day 16 of pregnancy
DP25: Day 25 of pregnancy
GnRH: Gonadotropin releasing hormone
GAPDH: Glyceraldehyde-3-phosphate dehydrogenase
IFNT: Interferon tau
IL: Interleukin
ISGs: Interferon stimulated genes
NF-κB: Nuclear factor kappa B
P4: Progesterone
TLRs: Toll-like receptors
TNF: Tumor necrosis factor

## References

[1] Deshmukh H, Way SS (2019). Immunological basis for recurrent fetal loss and pregnancy complications. Annu Rev Pathol.

[2] Ott TL (2020). Immunological detection of pregnancy: evidence for systemic immune modulation during early pregnancy in ruminants. Theriogenology.

[3] Forde N, Lonergan P (2017). Interferon-tau and fertility in ruminants. Reproduction.

[4] Bazer FW, Seo H, Wu G, Johnson GA (2020). Interferon tau: influences on growth and development of the conceptus. Theriogenology.

[5] Yang L, Li N, Zhang L, Bai J, Zhao Z, Wang Y (2020). Effects of early pregnancy on expression of interferon-stimulated gene 15, STAT1, OAS1, MX1, and IP-10 in ovine liver. Anim Sci J.

[6] Alak I, Hitit M, Kose M, Kaya MS, Ucar EH, Atli Z, Atli MO (2020). Relative abundance and localization of interferon-stimulated gene 15 mRNA transcript in intra- and extra-uterine tissues during the early stages of pregnancy in sheep. Anim Reprod Sci.

[7] Yang L, Liu B, Yan X, Zhang L, Gao F, Liu Z (2017). Expression of ISG15 in bone marrow during early pregnancy in ewes. Kafkas Univ Vet Fak Derg.

[8] Zhang L, Xue J, Wang Q, Lv W, Mi H, Liu Y, Yang L (2018). Changes in expression of ISG15, progesterone receptor and progesterone-induced blocking factor in ovine thymus during early pregnancy. Theriogenology.

[9] Yang L, Liu Y, Lv W, Wang P, Wang B, Xue J, Zhang L (2018). Expression of interferon-stimulated gene 15-kDa protein, cyclooxygenase (COX) 1, COX-2, aldo-keto reductase family 1, member B1, and prostaglandin E synthase in the spleen during early pregnancy in sheep. Anim Sci J.

[10] Wang Y, Han X, Zhang L, Cao N, Cao L, Yang L (2019). Early pregnancy induces expression of STAT1, OAS1 and CXCL10 in ovine spleen. Animals.

[11] Yang L, Wang Q, Liu Y, Zhang L, Lv W, Liu B (2019). Expression profiles of interferon-stimulated gene 15 and prostaglandin synthases in the ovine lymph nodes during early pregnancy. Mol Reprod Dev.

[12] Zhang L, Cao L, Yang F, Han X, Wang Y, Cao N, Yang L (2020). Relative abundance of interferon-stimulated genes STAT1, OAS1, CXCL10 and MX1 in ovine lymph nodes during early pregnancy. Anim Reprod Sci.

[13] Bellomo A, Gentek R, Bajénoff M, Baratin M (2018). Lymph node macrophages: scavengers, immune sentinels and trophic effectors. Cell Immunol.

[14] McLean JM, Mosley JG, Gibbs AC (1974). Changes in the thymus, spleen and lymph nodes during pregnancy and lactation in the rat. J Anat.

[15] Hetherington CM, Humber DP (1977). The effect of pregnancy on lymph node weight in the mouse. J Immunogenet.

[16] Kapovic M, Rukavina D (1991). Kinetics of lymphoproliferative responses of lymphocytes harvested from the uterine draining lymph nodes during pregnancy in rats. J Reprod Immunol.

[17] Yang L, Zang S, Bai Y, Yao X, Zhang L (2017). Effect of early pregnancy on the expression of progesterone receptor and progesterone-induced blocking factor in ovine lymph node. Theriogenology.

[18] Yang L, Wang P, Mi H, Lv W, Liu B, Du J, Zhang L (2019). Comparison of Th1 and Th2 cytokines production in ovine lymph nodes during early pregnancy. Theriogenology.

[19] Bai J, Zhang L, Zhao Z, Li N, Wang B, Yang L (2020). Expression of melatonin receptors and CD4 in the ovine thymus, lymph node, spleen and liver during early pregnancy. Immunology.

[20] Cao N, Cao L, Gao M, Wang H, Zhang L (2021). Yang L changes in mRNA and protein levels of gonadotropin releasing hormone and receptor in ovine thymus, lymph node, spleen, and liver during early pregnancy. Domest Anim Endocrinol.

[21] Hao S, Fang H, Fang S, Zhang T, Zhang L, Yang L (2022). Changes in nuclear factor kappa B components expression in the ovine spleen during early pregnancy. J Anim Feed Sci.

[22] Li Q, Verma IM (2002). NF-kappaB regulation in the immune system. Nat Rev Immunol.

[23] Ghosh S, Hayden MS (2012). Celebrating 25 years of NF-κB research. Immunol Rev.

[24] Feldman I, Feldman GM, Mobarak C, Dunkelberg JC, Leslie KK (2007). Identification of proteins within the nuclear factor-kappa B transcriptional complex including estrogen receptor-alpha. Am J Obstet Gynecol.

[25] Socha MW, Malinowski B, Puk O, Wartęga M, Stankiewicz M, Kazdepka-Ziemińska A, Wiciński M (2021). The role of NF-κB in uterine spiral arteries remodeling, insight into the cornerstone of preeclampsia. Int J Mol Sci.

[26] Ulkumen B, Artunc Ulkumen B, Batir MB, Pala HG, Vatansever S, Cam S (2019). Impact of pregnancy and glucocorticoid treatment on NF-κB and MUC5AC in mucosa of rat larynx. J Voice.

[27] Wang F, Wang Y, Wang R, Qiu H, Chen L (2016). Predictive value of maternal serum NF-κB p65 and sTREM-1 for subclinical chorioamnionitis in premature rupture of membranes. Am J Reprod Immunol.

[28] Sakowicz A (2018). The role of NFκB in the three stages of pregnancy - implantation, maintenance, and labour: a review article. BJOG.

[29] Cartwright T, Perkins ND, Wilson L, C. (2016). NFKB1: a suppressor of inflammation, ageing and cancer. FEBS J.

[30] Rahardjo B, Widjajanto E, Sujuti H, Keman K (2014). Different levels of IL-1α, IL-6, TNF-α, NF-κB and PPAR-γ in monocyte cultures exposed by plasma preeclampsia and normotensive pregnancy. Pregnancy Hypertens.

[31] Chapman NR, Europe-Finner GN, Robson SC (2004). Expression and deoxyribonucleic acid-binding activity of the nuclear factor kappaB family in the human myometrium during pregnancy and labor. J Clin Endocrinol Metab.

[32] Sun SC (2012). The noncanonical NF-κB pathway. Immunol Rev.

[33] Di Stefano V, Wang B, Parobchak N, Roche N, Rosen T. RelB/p52-mediated NF-κB signaling alters histone acetylation to increase the abundance of corticotropin-releasing hormone in human placenta. Sci Signal 2015;8:ra85. 10.1126/scisignal.aaa9806 26307012

[34] Yu LJ, Wang B, Parobchak N, Roche N, Rosen T (2015). STAT3 cooperates with the non-canonical NF-κB signaling to regulate pro-labor genes in the human placenta. Placenta.

[35] Wang B, Wang P, Parobchak N, Treff N, Tao X, Wang J, Rosen T (2019). Integrated RNA-seq and ChIP-seq analysis reveals a feed-forward loop regulating H3K9ac and key labor drivers in human placenta. Placenta.

[36] Valovka T, Hottiger MO (2011). p65 controls NF-κB activity by regulating cellular localization of IκBβ. Biochem J.

[37] Ersahin A, Acet M, Acet T, Yavuz Y (2016). Disturbed endometrial NF-κB expression in women with recurrent implantation failure. Eur Rev Med Pharmacol Sci.

[38] Hadfield KA, McCracken SA, Ashton AW, Nguyen TG, Morris JM (2011). Regulated suppression of NF-κB throughout pregnancy maintains a favourable cytokine environment necessary for pregnancy success. J Reprod Immunol.

[39] McCracken SA, Gallery E, Morris JM (2004). Pregnancy-specific down-regulation of NF-kappa B expression in T cells in humans is essential for the maintenance of the cytokine profile required for pregnancy success. J Immunol.

[40] Willard-Mack CL (2006). Normal structure, function, and histology of lymph nodes. Toxicol Pathol.

[41] Wang B, Palomares K, Parobchak N, Cece J, Rosen M, Nguyen A, Rosen T (2013). Glucocorticoid receptor signaling contributes to constitutive activation of the noncanonical NF-κB pathway in term human placenta. Mol Endocrinol.

[42] Wang XK, Agarwal M, Parobchak N, Rosen A, Vetrano AM, Srinivasan A, Wang B, Rosen T (2016). Mono-(2-Ethylhexyl) phthalate promotes pro-labor gene expression in the human placenta. PLoS One.

[43] Masat E, Gasparini C, Agostinis C, Bossi F, Radillo O, De Seta F, Tamassia N, Cassatella MA, Bulla R (2015). RelB activation in anti-inflammatory decidual endothelial cells: a master plan to avoid pregnancy failure?. Sci Rep.

[44] Sharfe N, Merico D, Karanxha A, Macdonald C, Dadi H, Ngan B, Herbrick JA, Roifman CM (2015). The effects of RelB deficiency on lymphocyte development and function. J Autoimmun.

[45] de Jesús TJ, Ramakrishnan P. NF-κB c-Rel dictates the inflammatory threshold by acting as a transcriptional repressor iScience. 2020;23:100876. 10.1016/j.isci.2020.100876 PMC7031323 32062419

[46] Sekiya Y, Yamamoto E, Niimi K, Nishino K, Nakamura K, Kotani T, Kajiyama H, Shibata K, Kikkawa F (2017). C-Rel promotes invasion of choriocarcinoma cells via PI3K/AKT signaling. Oncology.

[47] Cookson VJ, Chapman NR (2010). NF-kappaB function in the human myometrium during pregnancy and parturition. Histol Histopathol.

[48] Schmittgen TD, Livak KJ (2008). Analyzing real-time PCR data by the comparative C(T) method. Nat Protoc.

[49] Kandil D, Leiman G, Allegretta M, Trotman W, Pantanowitz L, Goulart R, Evans M (2007). Glypican-3 immunocytochemistry in liver fine-needle aspirates: a novel stain to assist in the differentiation of benign and malignant liver lesions. Cancer.

