# Supplementary figures and images for: Expression of nuclear factor kappa B in ovine maternal inguinal lymph nodes during early pregnancy
Source: BMC Vet Res. 2022 Jul 11;18:266. doi: 10.1186/s12917-022-03373-7 (PMC9275262; doi:10.1186/s12917-022-03373-7)

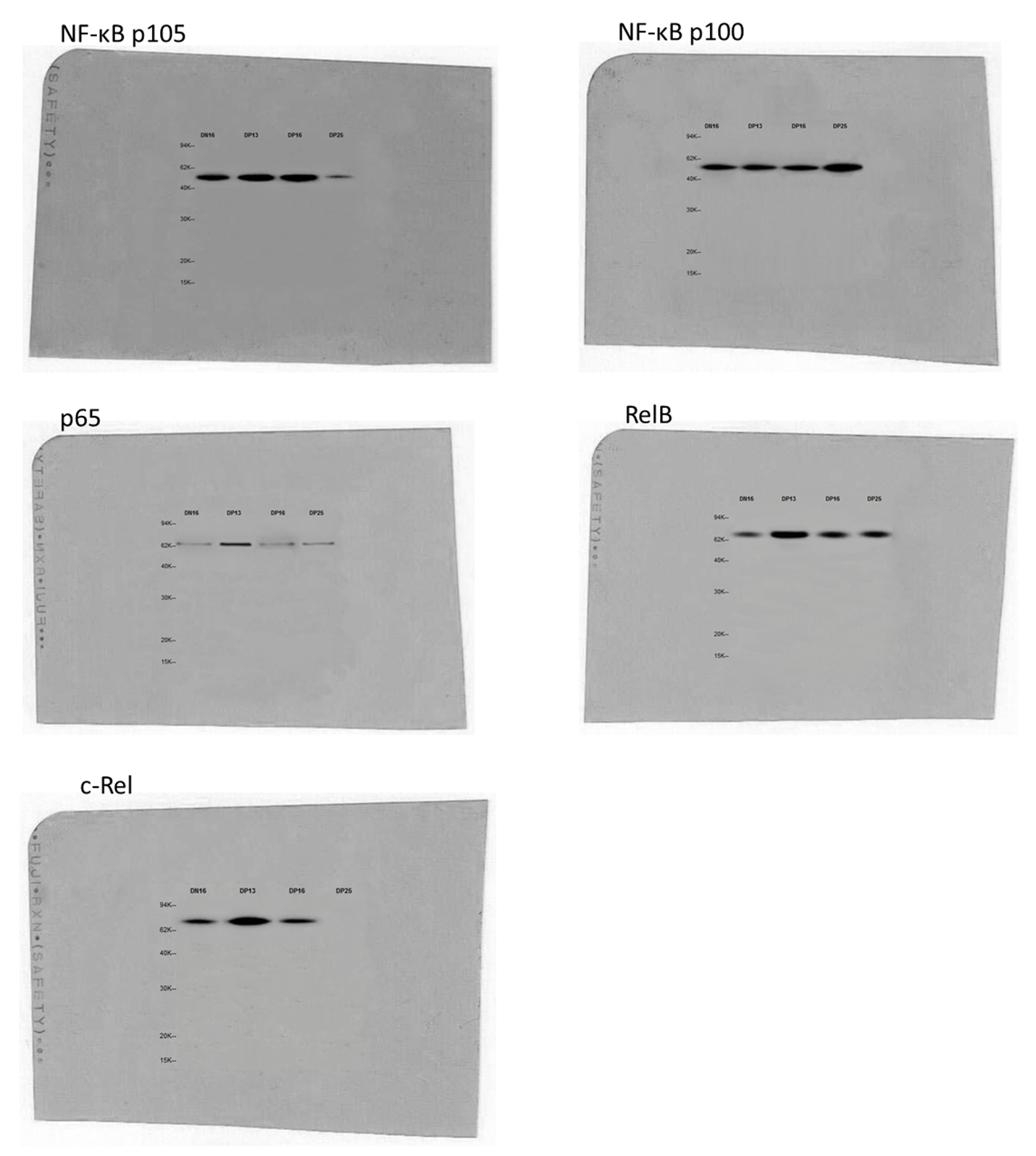

Supplement: Supplementary file 1 — Additional file 1. [file 12917_2022_3373_MOESM1_ESM.tif]
